# RawVegetable 2.0: Refining XL-MS Data Acquisition through Enhanced Quality Control

**DOI:** 10.1021/acs.jproteome.3c00791

**Published:** 2024-02-01

**Authors:** Louise Ulrich Kurt, Milan Avila Clasen, Ísis Venturi Biembengut, Max Ruwolt, Fan Liu, Fabio César Gozzo, Diogo Borges Lima, Paulo Costa Carvalho

**Affiliations:** † Laboratory for Structural and Computational Proteomics, Carlos Chagas Institute - Fiocruz Parana, Curitiba, Parana 81310-020, Brazil; ‡ Department of Chemical Biology, Leibniz - Forschungsinstitut für Molekulare Pharmakologie (FMP), Berlin 13125, Germany; § Dalton Mass Spectrometry Laboratory, Unicamp, Campinas, Sao Paulo 13083-970, Brazil

**Keywords:** cross-linking mass spectrometry, quality control, bioinformatics

## Abstract

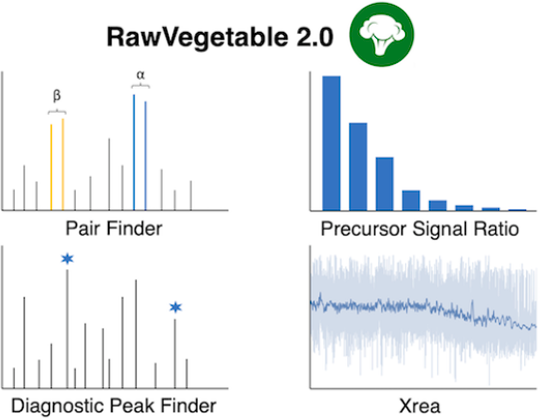

We present RawVegetable 2.0, a software tailored for assessing mass spectrometry data quality and fine-tuned for cross-linking mass spectrometry (XL-MS) applications. Building upon the capabilities of its predecessor, RawVegetable 2.0 introduces four main modules, each providing distinct and new functionalities: 1) Pair Finder, which identifies ion doublets characteristic of cleavable cross-linking experiments; 2) Diagnostic Peak Finder, which locates potential reporter ions associated with a specific cross-linker; 3) Precursor Signal Ratio, which computes the ratio between precursor intensity and the total signal in an MS/MS scan; and 4) Xrea, which evaluates spectral quality by analyzing the heterogeneity of peak intensities within a spectrum. These modules collectively streamline the process of optimizing mass spectrometry data acquisition for both Proteomics and XL-MS experiments. RawVegetable 2.0, along with a comprehensive tutorial is freely accessible for academic use at: http://patternlabforproteomics.org/rawvegetable2.

## Introduction

**Figure 1:**
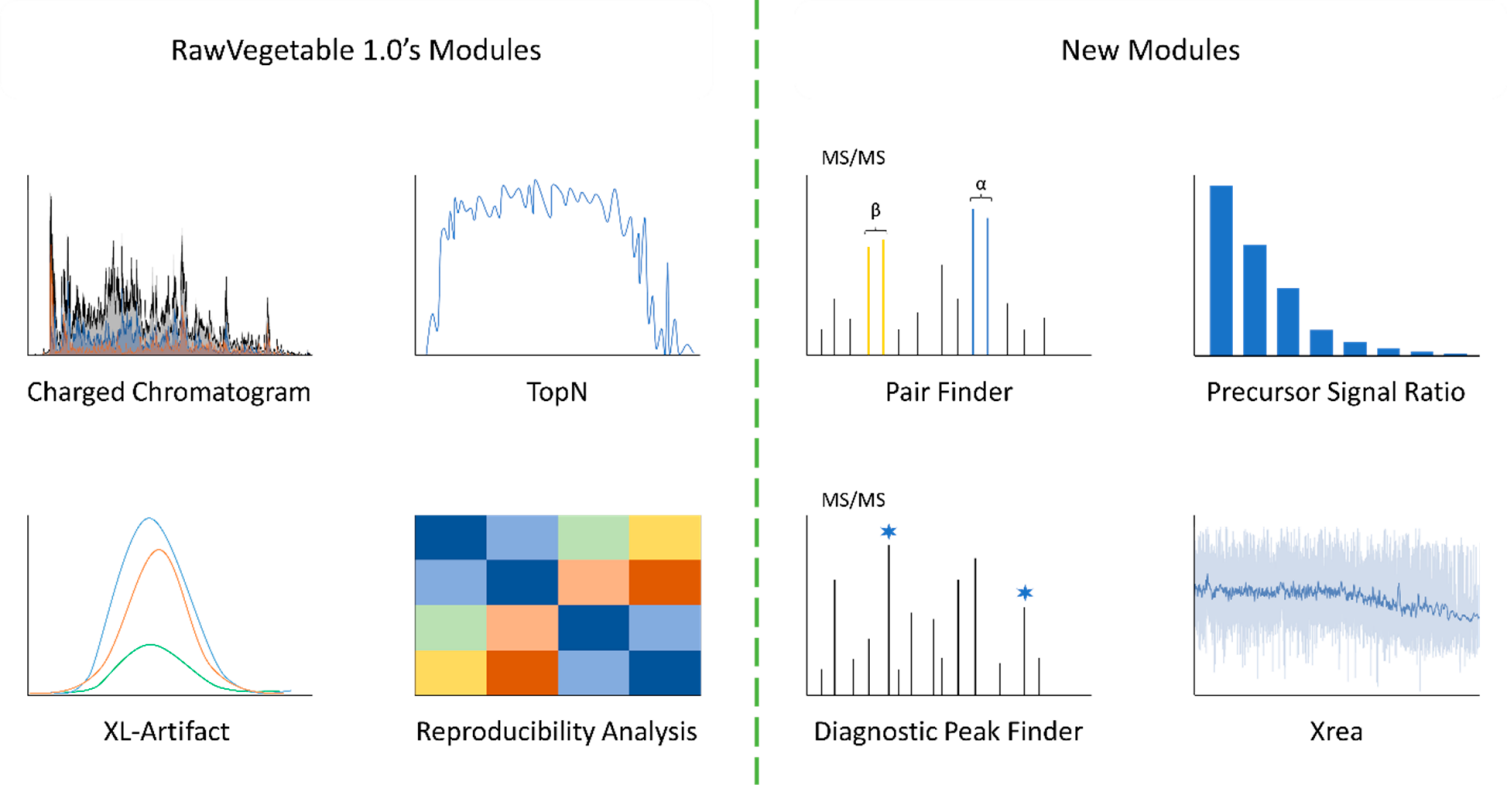
Overall view of RawVegetable 2.0s modules. From the previous version, we have the Charged Chromatogram and the TopN Estimation providing retention time vs precursor ion charges or number of MS/MS scans per duty cycle respectively, as well as the XL-Artifact module for flagging spurious peptide to peptide XL identifications, and the Reproducibility Analysis for observing quantitative changes across experimental/biological replicates. Exclusive to RawVegetable 2.0 we introduce the Pair Finder module for searching MS/MS data for doublet ion pairs from cleavable cross-linker reagents, the Precursor Signal Ratio for estimating the percentage of MS/MS precursor peaks consumed during fragmentation, the Diagnostic Peak Finder for searching for recurrent ions across all scans in a run, and finally the Xrea module, wherein a top-level view of average MS/MS spectrum quality is provided across the whole data acquisition.

Cross-linking mass spectrometry (XL-MS) stands out as a potent method for probing protein–protein interactions (PPIs) within exceedingly complex samples.^1^ Cross-linking reagents are small molecules consisting of a spacer arm with a reactive site on each end. Upon introduction into a protein sample, these reactive sites selectively form covalent bonds with specific amino acids. This process creates bridges between amino acids located in the proximity of the sample, constrained by the length of the spacer arm. The sample, now containing cross-linked proteins, can then be denatured and digested as in shotgun proteomics protocols. This results in a peptide mixture that includes both linear, unmodified peptides and three distinct types of cross-linked molecules. The first type involves peptides where one end of the cross-linking (XL) reagent is linked to an amino acid, while the other end remains unlinked, commonly termed dead-end XL identifications or monolinks. The second type comprises peptides in which both ends of the same XL reagent are linked to different amino acids within the peptide, known as intralink XL identifications or looplinks. Lastly, species where two distinct peptides are linked, and thus can be identified in the same chimeric MS/MS spectrum, are referred to as interlinks.^2−4^ The naming conventions for interlink XL identifications are that the one with the higher number of amino acids be called α peptide, with the second being β peptide. In the case of equal lengths, the α is the first peptide alphabetically. XL-MS then typically relies on liquid chromatography coupled with tandem mass spectrometry, often with the added steps of sample fractionation and enrichment to reach the lower-abundance cross-linked species, producing hundreds of thousands of mass spectra. The several additional steps, in sample preparation and data generation, are linked with increased experimental variability and thus exacerbate the need for specialized computational environments for comprehensive data analysis. In such environments, quality control is an essential step of a mass spectrometry experiment since it is a highly complex process, thus containing a number of moments where variability can be introduced, both experimentally and computationally. As a few examples, slight shifts during the digestion of proteins, an uncalibrated mass spectrometer, a faulty or old column can all cause alterations in what was expected from an experiment.^5^ Moreover, choosing the right method for the mass spectrometer to operate is fundamental in obtaining the best results from the analysis and establishing protocols to be followed. In XL-MS experiments, the additional steps of cross-linking the proteins and enrichment will all contribute to increased variability as the complexity of the samples and of the experiment itself increases. A better understanding of the enriched chromatographic fractions is one of the first key steps for cross-linking peptide identification, as selecting only those fractions that are most likely to have more cross-linked and fewer linear peptides can greatly save time in the analysis. With this in mind, we originally developed RawVegetable 1.0,^6^ which, in analogy to Vast Scientific’s RawMeat, contains several modules for the assessment of LC/MS runs, with special focus on XL-MS experiments (Figure 1). Among these modules, we highlight the Charge State Chromatogram and the TopN Density Estimation, both of which provide useful information about the acquisition efficiency and can help with gradient optimization. Briefly, the charge-state chromatogram consists of deconvoluting the MS spectra and displaying only the summed signal of the species of a single charge, identifying regions where highly charged species are eluting. The TopN Density Estimation entails the calculation of the distribution of MS/MS scans per MS event throughout the chromatography run, which allows identifying regions of possible over- or under-sampling.

The numerous feature requests from the proteomic community have guided the development of several new features in RawVegetable 2.0, culminating in version 2.0 (Figure 1). Among these enhancements, the Xrea module provides an overview of spectra quality, with regards to overall intensity and ion distribution. The Precursor Signal Ratio module aims at investigating precursor ion fragmentation, to evaluate the effect of different collision energies on produced peptide fragments. And finally, the Pair Finder module, which is tailored specifically for cleavable cross-linker reagents, allows users to check for the formation of the ion doublets vital for identification of cross-linked species, while the Diagnostic Peak Finder looks for reporter ions that may be generated across all spectra, which is of particular interest for cross-linked samples.

## Methods

### Pair Finder

This module assesses each MS/MS spectrum in each run to search for unique peak doublets indicative of cleavable cross-linking peptide species. These doublets emerge when a cleavable cross-linker undergoes asymmetrical cleavage, leading to the formation of two distinct sets of peptide ion pairs. Each set emerges from one of the peptides (designated by α and β) previously joined by the cross-linker. The key feature of these doublets is their consistent delta mass, a direct consequence of the cross-linker’s asymmetrical cleavage. This delta mass serves as a distinctive marker, revealing the individual masses of the α and β peptides.^7^ The Pair Finder algorithm works by looking for a pair of peaks with a difference in mass of such delta; once a pair is found (either α or β peptide), it looks for its complementary one based on the precursor and cross-linker mass. If these two pairs are found, which we refer to as an ion doublet, then the chances of the spectra belonging to a cross-linked peptide are quite high.

In order to minimize false hits, the Pair Finder algorithm first deconvolutes the tandem mass spectra by using Y.A.D.A. 3.0,^8^ thus mostly eliminating noise peaks and reducing the search to singly charged monoisotopic ions. Since this can make the search more stringent, all deconvolution parameters are accessible to the user, should a special condition be required to not use our default parameters.

Once the algorithm has searched all spectra for ion doublets, a percentage of scans where the doublets were found will be reported to the user. This information will be displayed in different plots: a bar plot that compares the percentages of all loaded files as well as boxplots representing the distribution of the summed intensities of each pair in the doublets for each file; and a line plot displaying regions of the chromatographic run where more doublets were found. A spectrum explorer is made available for visualizing the doublets found in individual scans. The user can also export only the spectra where such doublets were found, possibly reducing the search time in subsequent analyses for specialized XL-MS search engines.

### Diagnostic Peak Finder

While the Pair Finder looks for specific doublet peaks based on known masses coming from the cleavable cross-linker, here, the algorithm traverses all MS/MS ions in search of recurrent peaks that may be novel products of peptide fragmentation, potentially locating previously unknown reporter ions specific to the sample at hand. We recall that reporter ions are ions that could indicate that the spectrum has originated from cross-linked peptides.^10^ This search can be performed either on all MS/MS spectra or only on a subset of identifications (either of linear or cross-linked peptides). Once more frequent peaks have been detected, all spectra containing them can be exported. Considering that the search engine will examine the exported set of spectra, the processing time is expected to be decreased in comparison to a comprehensive search involving all of the spectra in the RAW file. Moreover, the chance of finding false positives will be lower, thus, increasing the overall sensitivity of the search engine.

Furthermore, the Diagnostic Peak Finder can also be used to search for possible known diagnostic ions generated by the cross-linker under HCD and CID fragmentation conditions,^9^ but their known masses should be reported by the user. While the focus is on XL-MS, it is important to note that this feature is not limited to that and it can be used to search for any reporter ion of interest; for example, even to find only the spectra that contain isobaric labels, such as TMT^11^ or iTRAQ.^12^

### Precursor Signal Ratio

This feature consists of calculating the ratio between the intensity of the precursor ion from an MS/MS scan and the total signal from said scan. In this regard, one can evaluate the spectral composition by determining the proportion of the precursor peak compared with the successfully fragmented ions. A ratio close to zero indicates that most of the precursor ion has been consumed when generating the fragments, and thus a greater degree of successful fragmentation in the spectrum. This is especially important in cleavable cross-linking experiments because many search engines depend on the formation of the ion doublets (as described in the Pair Finder algorithm), and a bad fragmentation of the precursor will not lead to the formation of these peaks and subsequent identification of such spectrum. This is also true for linear peptides, which hinge on successful fragmentation to identify them.

### Xrea

This score has been introduced by Na and Paek^13^ as an indicator of the quality of a tandem mass spectrum (MS/MS). Briefly, it is calculated based on the heterogeneity of peak intensities within a spectrum, as lower quality spectra tend to produce many peaks of similar intensity. This score ranges from 0 to 1, the higher the score, the more heterogeneous the spectrum, possibly indicating an increase in its quality. RawVegetable 2.0 displays a plot of the Xrea over the chromatography retention time, with also a smoothed trendline being displayed, which greatly helps identify regions of the run where the best spectra are being produced. An example of the plot generated by RawVegetable 2.0 can be seen in the Supporting Information—Figure S1.

### General Updates

For the already existing modules in RawVegetable 2.0, such as the XL-Artifact Search and the Reproducibility Analysis, this new version accepts other results besides SIM-XL^14^ and PatternLab for Proteomics.^15^ The software now features a versatile reader that supports both *.csv and *.txt file formats. This functionality is designed to streamline the process of handling cross-link identifications, where users need to specify key information such as sequence, *m*/*z*, charge, and other relevant parameters within their data files. In this regard, users can load text files and designate columns containing essential details. The user-friendly interface, as depicted in Figure S22 in the Supporting Information, provides a convenient screenshot illustrating how users can easily configure and customize the identification columns. Importantly, not all information is required for every analysis, allowing users to tailor their data processing to their specific needs. All functionality details can be accessed on the software web site.

To address the evolving throughput of the new mass spectrometers, we have also updated our deconvolution routines with Y.A.D.A. 3.0, that stands out as a fast and less error prone deconvolution algorithm.^8^

RawVegetable 2.0 is also capable of analyzing spectrum data originating from timsTOF® (Bruker) and Astral® (Thermo Fisher Scientific) spectrometers.

## Case Studies

### Identifying Optimal Enrichment Strategies in Cross-Linking Experiments with the Pair Finder Module

**Figure 2:**
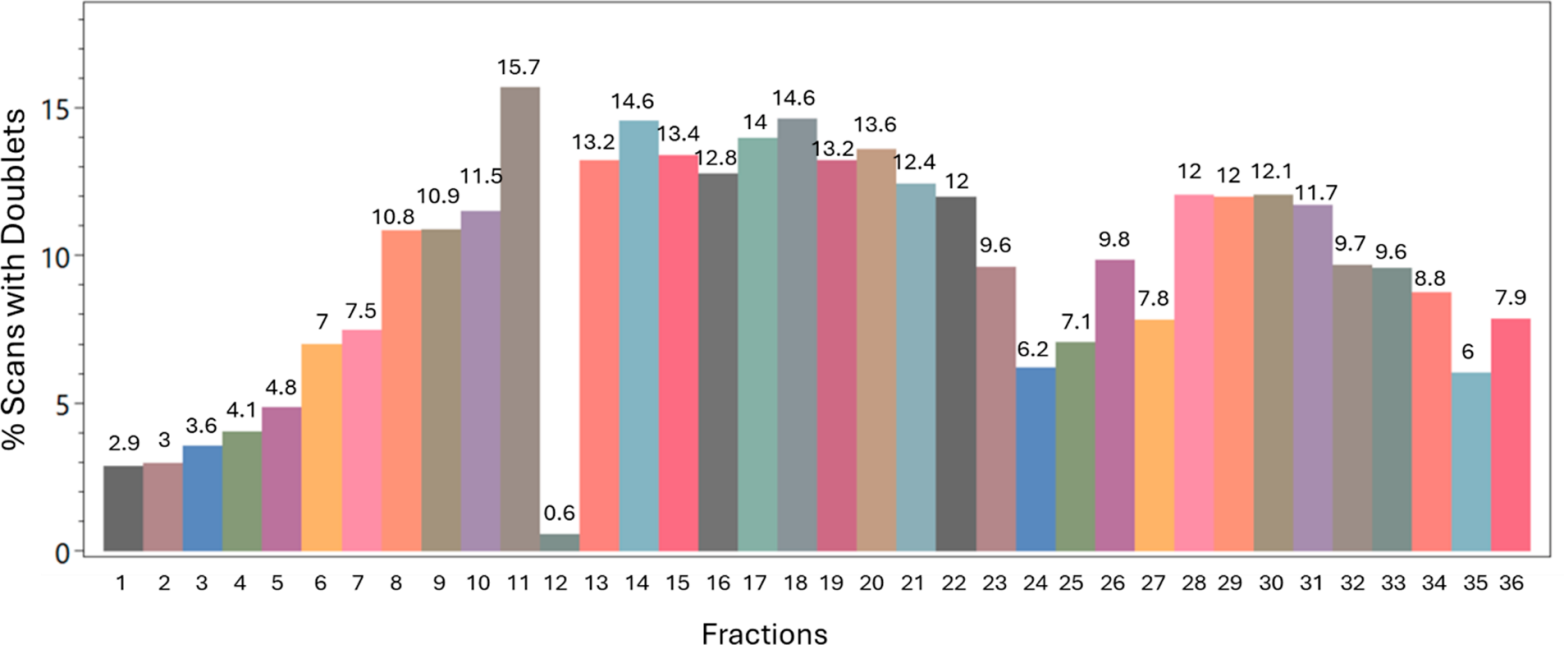
Histogram plot produced by the Pair Finder module, showing the percentage, per SCX fraction, of MS/MS spectra with the ion doublets characteristic of peptide species cross-linked by DSSO. The first fractions, where smaller species are expected to elute, produced a smaller number of ion doublets. On the plot, each bar represents a fraction, with the numbers on top reporting the percentage of scans with doublets.

As a proof of concept to demonstrate the usefulness of the Pair Finder module, we analyzed data from^16^ (ProteomeXchange PXD031911), which makes use of XL-MS, and specifically, the DSSO cross-linker reagent^17^ to study the interactome of intact human cytomegalovirus virions. In their experiments, the authors performed SCX fractionation, which generated 36 different fractions to be run on the mass spectrometer. The summary of the raw files used to test RawVegetable 2.0 as well as the detailed results can be found on the Supporting Information—Table S1 and Figure S2. In this study, later fractions would have more cross-linkers; however, being able to decide early which fractions should be used for the cross-link search, and even exporting only the necessary spectra, could save a great amount of time. Our tests with this data set indeed showed that later fractions had an increased number of cleavable pairs being formed, suggesting that from the 36 fractions generated, at least 10 had less than 7% of the spectra with doublets, which would not generate a good amount of cross-link identifications in comparison to linear peptides, possibly hindering FDR calculation and not providing enough information to answer the biological questions of interest. Another interesting point is, the file for one of the fractions (12) was incorrectly loaded into the software by the authors and RawVegetable 2.0 was successfully able to identify it as having a low number of pairs, possibly flagging it as problematic, as can be seen in Figure 2.

**Figure 3:**
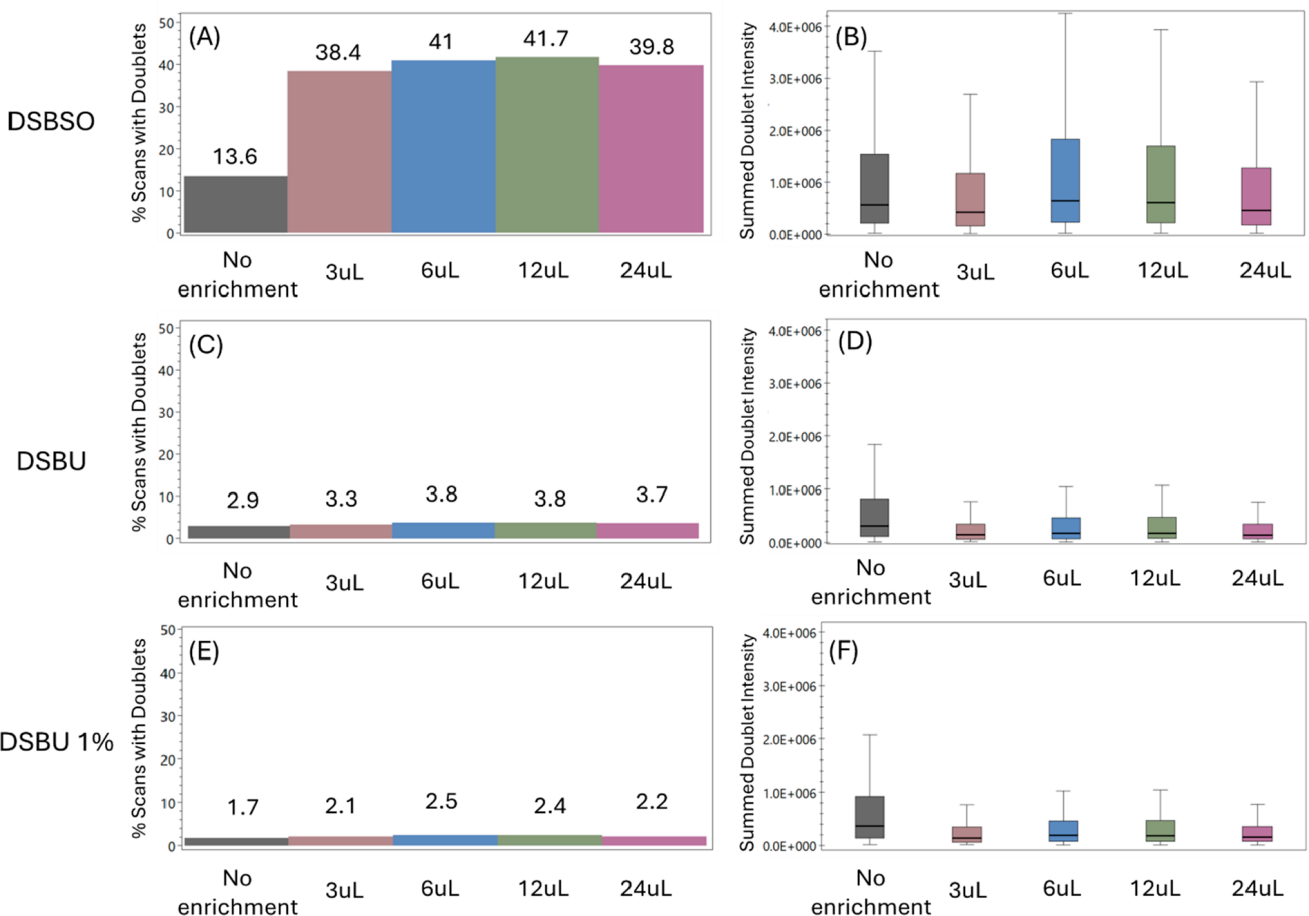
Pair Finder results for DSBSO enrichment data set. (A) represents the percentage of scans which had the DSBSO cross-link doublets across the entire run in each enrichment condition. Panels (C) and (E) are repeats of the same analysis but using the masses for the DSBU reagent with no intensity cutoff and with a 1% intensity cutoff, respectively. The specific scan percentage for each condition is shown on top of the individual bars. (B–F) Distribution of the summed intensities of the doublets found in each scan in the form of boxplots for the same parameters. All plots have the same scale for the different tests.

We executed Pair Finder on this data set with stringent deconvolution parameters and no intensity cutoff. Interestingly, the results show that the most successful runs yielded approximately 15% of the MS/MS spectra with the expected pairs. In order to test if cross-linkers with extra enrichment steps, such as DSBSO, could produce a higher percentage of scans with the doublets, a part of the data set from^18^ (ProteomeXchange PXD016963) was analyzed. In this paper, the authors introduce a method to enrich samples cross-linked with the reagent Azide-A-DSBSO using DBCO beads and test their protocols in a series of experiments with recombinant Cas9 and *Escherichia coli* ribosome. We selected five samples from the whole data set, specifically the experiments with Cas9 where the authors test various amounts of beads to determine the optimal condition for the enrichment. A summary of the files analyzed, as well as detailed results from the Pair Finder module, can be found in the Supporting Information—Tables S2–S4. The results from Pair Finder indicated that the sample with no enrichment showed only 13.6% of spectra with doublets (Figure 3A), while the best enrichment configuration (12 μL) got 41.7% (Figure 3A). This is also in exact agreement with what the original paper shows pertaining to the number of cross-links identified in each configuration. Interestingly, the mean of the summed doublet intensity between the no enrichment and the best enrichment samples was very similar, as can be seen in Figure 3B.

To estimate the percentage of the scans resulting from peaks that match doublet ion signatures by chance, we have also intentionally analyzed this DSBSO data set with incorrect masses (the masses of DSBU^19^ were used—Figure 3C), and the percentages of spectra with pairs decreases to around 3.5%, and the overall summed intensity of the pairs also decreases (Figure 3D). If an intensity cutoff of 1% of the maximum signal is applied, this number goes even lower to around 2% (Figures 3E and 3F).

### Using RawVegetable 2.0 to Optimize Collision Energy for Peptide Dissociation

**Figure 4:**
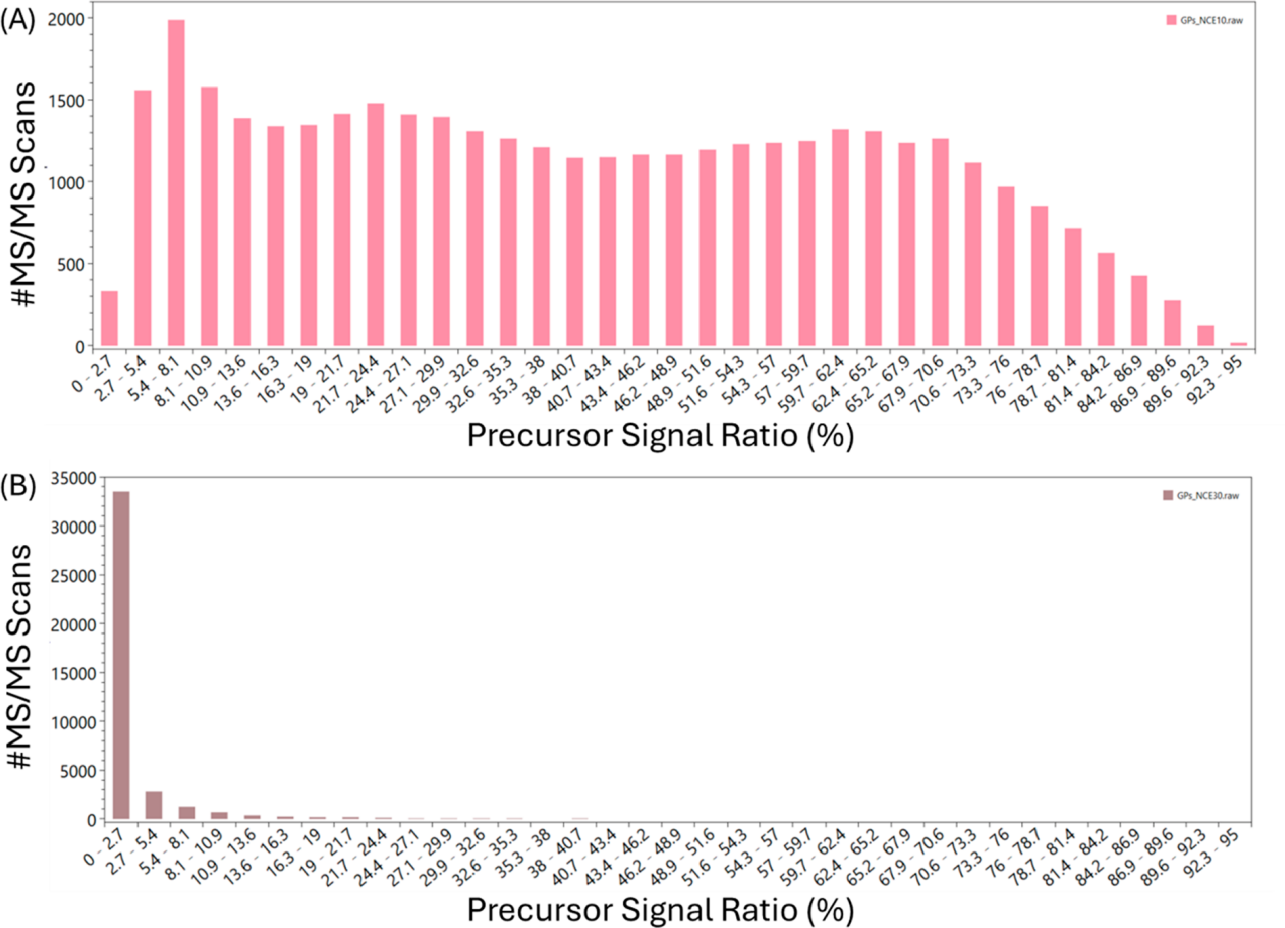
An example of two different distributions of the percentage of precursor signal left in the N-glycopeptides data set. Panel (A) is the distribution for the run that used an NCE of 10%, while panel (B) is for NCE 30%.

Here, we show how our software could be used to optimize the Normalized Collision Energy (NCE) for peptide dissociation. For this purpose, we tested two different data sets. The first one is from^20^ (ProteomeXchange PXD016865), which describes how different NCEs, and specifically, how a combination of energies in a stepped experiment can affect the results generated when analyzing N-glycopeptides, as the peptide backbone and the N-glycan moieties fragment at different NCEs. We only analyzed the data from the separate NCE experiments (nine NCEs ranging from 10% to 50% in increments of 5 units) and ran them on RawVegetable 2.0. Usually, for shotgun proteomics, the mass spectral peak decurrent from the precursor ion should be minimal; thus, a high intensity peak can be generally associated with poor fragmentation. Notably a very high NCE could cause the peptide to be overfragmented, which may impair peptide spectrum matching. The expected pattern for the distribution of precursor signal ratio across all scans would be an exponential decrease, where most scans have almost no precursor left (a ratio close to 0%), a few still have the precursor making up to 10–15% of the total signal of the spectrum, and almost no scans have a ratio higher than 20–25%. For the N-glycopeptides data set, in the NCEs 10% until 20%, the fragmentation of the peptides is quite poor, as exemplified by Figure 4A, which shows the precursor signal ratio distribution for NCE 10%. The expected aforementioned pattern that indicates a successful fragmentation is seen for NCEs 25% and 30% (Figure 4B), which are in fact the separate collision energies that gave the best results in the paper. (The complete analysis is shown in the Supporting Information—Figures S3–S11.)

**Figure 5:**
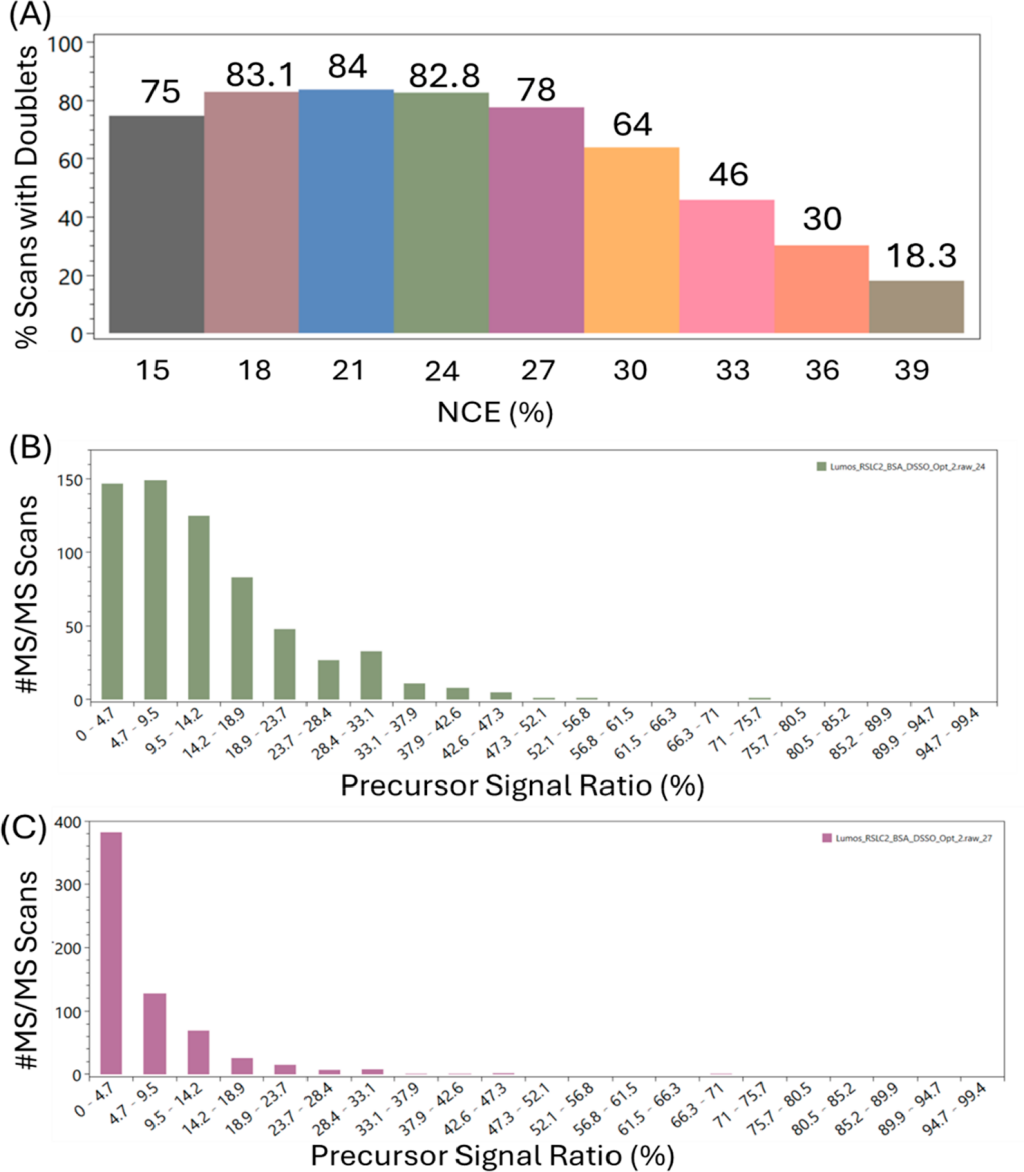
RawVegetable 2.0s results for the collision energy analysis in the cross-link data set from ref (21). (A) Pair Finder results for each different NCE used in the form of a bar plot of the percentage of scans with a doublet. (B) Precursor Signal Ratio distribution for the scans with NCE 24% and (C) Precursor Signal Ratio distribution for the scans with NCE = 27%.

We additionally examined a data set from ref (21) (ProteomeXchange PXD011861), which is focused on a cross-linked peptide experiment that explored various strategies for optimizing the fragmentation of peptides cross-linked with cleavable reagents. One of the experiments within this data set was subjected to detailed analysis, involving the acquisition of BSA cross-linked peptides with the reagent DSSO across nine distinct NCEs ranging from 15% to 39% with three-unit increments, all within a single run. We separated the spectra from each NCE in different files in order to load them into RawVegetable 2.0. A summary containing the name of the file used, as well as the NCEs it was separated into, and the detailed results for the Pair Finder module can be seen in the Supporting Information—Table S5. Initial results using the Precursor Signal Ratio module would suggest that the NCEs 27% and higher would be the most appropriate ones, as they produce the expected pattern for the histogram (Figure 5B shows an example of a lower NCE, 24%, which is still not good enough for peptide fragmentation, while Figure 5C shows the histogram for 27%, where the distribution of the precursor signal ratio is good). However, when the Pair Finder module is used, we noticed that from NCE 24% onward, the generation of doublets decreases, with more drastic changes after the 30%. So, in this case, we recommend using both modules in combination to determine the best NCE for the experiment, or even for determining a good range for stepped collision runs. For peptides cross-linked with cleavable reagents the peptides need to fragment enough to form the peaks necessary for the identification, but not so much so that you lose the peaks for the doublets. In this case, the Precursor Signal Ratio alone would not be enough to give you information on the quality of the run, and so running the Pair Finder module as well would be ideal in order to find a good compromise between the collision energies that give the best fragmentation without losing the doublets generated. For this data set, analyzing just the plots from RawVegetable 2.0, we noticed that the best collision energy is around 27%, which would be a good trade-off between having a good precursor ratio distribution, while maintaining an acceptable percentage of scans with doublets, as can be seen in Figures 5A and 5C. (The complete analysis is shown in the Supporting Information—Figures S12–S21.) This agrees with the results from the original paper, where the experiment with NCE = 27% gave the XL identifications with the highest score.

## Results and Discussion

Here we present RawVegetable 2.0, an updated version of our software for quality control of XL-MS data. This software provides a suite of modules to aid in improving mass spectrometry data acquisition, with a focus on XL-MS data sets. We showed how using RawVegetable 2.0s Pair Finder module can provide an overview of SCX fractionated data, locating the best range of fractions; this can be especially helpful in large experiments, to preselect fractions for potentially costly analysis, and also for pinpointing problematic files. With the use of the Precursor Signal Ratio module, we were able to observe the effects of increases to NCE on peptide fragmentation, which, when used together with the Pair Finder module, provided a clear strategy for obtaining an optimal set of parameters for cleavable cross-linker searches. These are all particularities of the data sets we analyzed that might potentially take a long time to evaluate, or even notice, but become simple with RawVegetable 2.0.

In the current XL-MS landscape, the absence of characteristic ion doublets from cleavable XL reagents can make certain data sets virtually unsearchable by most search engines. Furthermore, determining the optimal parameters for fragmenting cross-linked peptides often remains elusive, a challenge compounded as the field progresses toward more intricate experimental setups, including the use of TMT tags, diverse cleavable groups, or more complex reagents. For many mass spectrometry platforms and laboratories, the significance of these issues may only surface after extensive investment in mass spectrometer time and computational resources, underscoring the essential role of quality control, particularly given the heightened demands of XL-MS compared with traditional proteomics. As such, we anticipate that RawVegetable 2.0, adept for both bottom-up proteomics and specifically fine-tuned for XL-MS data, stands as a valuable asset to be included in any XL-MS laboratory’s repertoire.

## Data Availability

RawVegetable 2.0, accompanied by a comprehensive tutorial, is freely accessible for academic use at: http://patternlabforproteomics.org/rawvegetable2.

## Supporting Information Available

Supplementary Methods: Figure S1. The results from the Xrea module for two files from the DSBSO enrichment case study, specifically, the 12 μL of beads condition. Figure S2. Distribution of the doublets summed intensities in the form of boxplots for each fraction. Figure S3. Distribution of the percentage of precursor signal left in the MS2 scans from the experiment with NCE 10%. Figure S4. Distribution of the percentage of precursor signal left in the MS2 scans from the experiment with NCE 15%. Figure S5. Distribution of the percentage of precursor signal left in the MS2 scans from the experiment with NCE 20%. Figure S6. Distribution of the percentage of precursor signal left in the MS2 scans from the experiment with NCE 25%. Figure S7. Distribution of the percentage of precursor signal left in the MS2 scans from the experiment with NCE 30%. Figure S8. Distribution of the percentage of precursor signal left in the MS2 scans from the experiment with NCE 35%. Figure S9. Distribution of the percentage of precursor signal left in the MS2 scans from the experiment with NCE 40%. Figure S10. Distribution of the percentage of precursor signal left in the MS2 scans from the experiment with NCE 45%. Figure S11. Distribution of the percentage of precursor signal left in the MS2 scans from the experiment with NCE 50%. Figure S12. Distribution of the doublets summed intensities in the form of boxplots for each NCE. Figure S13. Distribution of the percentage of precursor signal left in the MS2 scans from the experiment with NCE 15%. Figure S14. Distribution of the percentage of precursor signal left in the MS2 scans from the experiment with NCE 18%. Figure S15. Distribution of the percentage of precursor signal left in the MS2 scans from the experiment with NCE 21%. Figure S16. Distribution of the percentage of precursor signal left in the MS2 scans from the experiment with NCE 24%. Figure S17. Distribution of the percentage of precursor signal left in the MS2 scans from the experiment with NCE 27%. Figure S18. Distribution of the percentage of precursor signal left in the MS2 scans from the experiment with NCE 30%. Figure S19. Distribution of the percentage of precursor signal left in the MS2 scans from the experiment with NCE 33%. Figure S20. Distribution of the percentage of precursor signal left in the MS2 scans from the experiment with NCE 36%. Figure S21. Distribution of the percentage of precursor signal left in the MS2 scans from the experiment with NCE 39%. Figure S22. Graphical user interface for loading identification results. The software now features a versatile reader that supports both *.csv and *.txt file formats. Table S1. Summary of the Pair Finder results for the SCX Fractionation case study. Table S2. Summary of the Pair Finder analysis for the enrichment data set, with DSBSO as the selected cross-linker. Table S3. Summary of the Pair Finder analysis for the enrichment data set, with DSBU as the selected cross-linker. Table S4. Summary of the Pair Finder analysis for the enrichment data set, with DSBU as the selected cross-linker and an intensity cutoff of 1%. Table S5. Summary of the Pair Finder results for the different NCEs applied. (PDF)
