# Supplementary material for: RawVegetable 2.0: Refining XL-MS Data Acquisition through Enhanced Quality Control
Source: J Proteome Res. 2024 Feb 1;23(8):3141–8. doi: 10.1021/acs.jproteome.3c00791 (PMC11301677; doi:10.1021/acs.jproteome.3c00791)
Supplement: Supplementary file 1 — pr3c00791_si_001.pdf [file pr3c00791_si_001.pdf]

RawVegetable 2.0 – Refining XL-MS data acquisition through enhanced quality control

Louise Ulrich Kurt[1], Milan Avila Clasen[1], Ísis Venturi Biembengut[1], Max Ruwolt[2], Fan Liu[2], Fabio César Gozzo[3], Diogo Borges Lima[2*], Paulo Costa Carvalho[1*]

[1] Laboratory for Structural and Computational Proteomics, Carlos Chagas Institute, Fiocruz-Parana 81310-020, Brazil
[2] Department of Chemical Biology, Leibniz - Forschungsinstitut für Molekulare Pharmakologie (FMP), Berlin, Germany
[3] Dalton Mass Spectrometry Laboratory, Unicamp, Campinas 13083-970, Brazil

*Correspondence authors: diogobor@gmail.com (D.B.L.); paulo@pcarvalho.com (P.C.C.)

Supplementary Material

**Figure S1**	The results from the Xrea module for two files from the DSBSO enrichment case study, specifically, the 12µL of beads condition.
**Figure S2**	Distribution of the doublets summed intensities in the form of boxplots for each fraction.
**Figure S3**	Distribution of the percentage of precursor signal left in the MS2 scans from the experiment with NCE 10%.
**Figure S4**	Distribution of the percentage of precursor signal left in the MS2 scans from the experiment with NCE 15%.
**Figure S5**	Distribution of the percentage of precursor signal left in the MS2 scans from the experiment with NCE 20%.
**Figure S6**	Distribution of the percentage of precursor signal left in the MS2 scans from the experiment with NCE 25%.
**Figure S7**	Distribution of the percentage of precursor signal left in the MS2 scans from the experiment with NCE 30%.
**Figure S8**	Distribution of the percentage of precursor signal left in the MS2 scans from the experiment with NCE 35%.
**Figure S9**	Distribution of the percentage of precursor signal left in the MS2 scans from the experiment with NCE 40%.
**Figure S10**	Distribution of the percentage of precursor signal left in the MS2 scans from the experiment with NCE 45%.
**Figure S11**	Distribution of the percentage of precursor signal left in the MS2 scans from the experiment with NCE 50%.
**Figure S12**	Distribution of the doublets summed intensities in the form of boxplots for each NCE.
**Figure S13**	Distribution of the percentage of precursor signal left in the MS2 scans from the experiment with NCE 15%.
**Figure S14**	Distribution of the percentage of precursor signal left in the MS2 scans from the experiment with NCE 18%.
**Figure S15**	Distribution of the percentage of precursor signal left in the MS2 scans from the experiment with NCE 21%.
**Figure S16**	Distribution of the percentage of precursor signal left in the MS2 scans from the experiment with NCE 24%.
**Figure S17**	Distribution of the percentage of precursor signal left in the MS2 scans from the experiment with NCE 27%.

**Figure S18**	Distribution of the percentage of precursor signal left in the MS2 scans from the experiment with NCE 30%.
**Figure S19**	Distribution of the percentage of precursor signal left in the MS2 scans from the experiment with NCE 33%.
**Figure S20**	Distribution of the percentage of precursor signal left in the MS2 scans from the experiment with NCE 36%.
**Figure S21**	Distribution of the percentage of precursor signal left in the MS2 scans from the experiment with NCE 39%.
**Figure S22**	Graphical user interface for loading identification results. The software now features a versatile reader that supports both *.csv and *.txt file formats.
**Table S1**	Summary of the Pair Finder results for the SCX Fractionation case study.
**Table S2**	Summary of the Pair Finder analysis for the enrichment dataset, with DSBSO as the selected cross-linker.
**Table S3**	Summary of the Pair Finder analysis for the enrichment dataset, with DSBU as the selected cross-linker.
**Table S4**	Summary of the Pair Finder analysis for the enrichment dataset, with DSBU as the selected cross-linker and an intensity cut-off of 1%.
**Table S5**	Summary of the Pair Finder results for the different NCEs applied.

1. Xrea Module Example

To exemplify how the results from the Xrea module are reported, we loaded one of the files from the DSBSO enrichment case study into RawVegetable 2.0. The Xrea score ranges from 0 to 1 and it is assigned to each MS/MS scan in a run (which would be the lighter orange curve in Figure S21), but to provide a better overview of the experiment, RawVegetable 2.0 provides a smoothed curve as well (the darker orange curve in Figure S1), which is calculated based on the Savitzky-Golay algorithm[1,2] and can have its parameters adjusted.

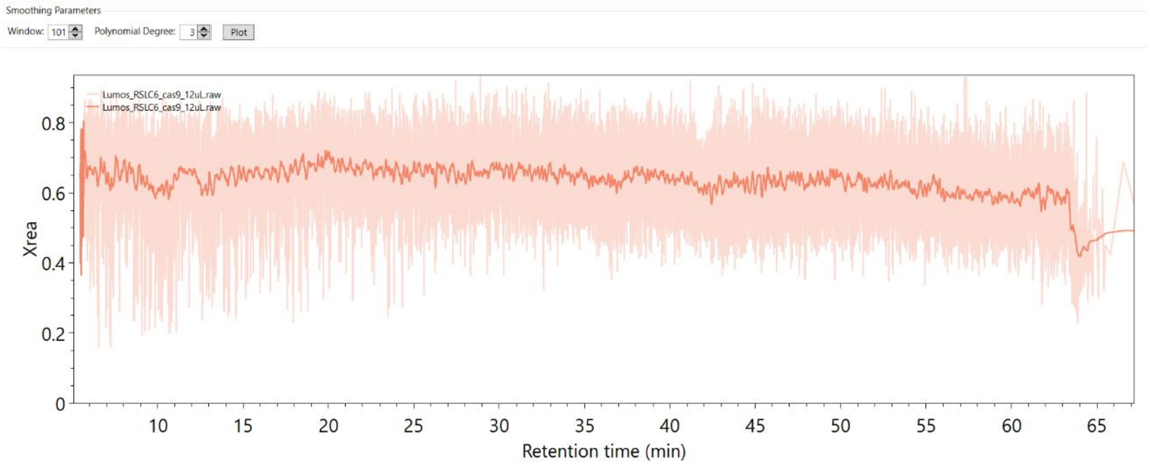

Figure S1. The results from the Xrea module for two files from the DSBSO enrichment case study, specifically, the 12μL of beads condition.

2. SCX Fractionation case study

The dataset used as a case study for the Pair Finder module (ProteomeXchange PXD031911) contained 36 files, which were run in RawVegetable 2.0 with the cross-linker DSSO. Table S1 contains the number of scans which had a doublet identified, as well as the total number of scans and the percentage of scans with doublets for each file analysed. Figure S2 displays the distribution of the summed intensity of the doublets for each fraction being analysed.

Table S1. Summary of the Pair Finder results for each fraction.

Fraction	File	#Scans with pairs	#Scans	%Pairs
1	L1_20210907_BB_HCMVVir_XL5mmb4_B1.raw	609	22066	2.882262304
2	L1_20210907_BB_HCMVVir_XL5mmb4_B2.raw	946	33836	2.990897269
3	L1_20210907_BB_HCMVVir_XL5mmb4_B3.raw	919	27190	3.556454579
4	L1_20210907_BB_HCMVVir_XL5mmb4_B4.raw	908	23805	4.062171813
5	L1_20210907_BB_HCMVVir_XL5mmb4_B5.raw	907	19915	4.855636455
6	L1_20210907_BB_HCMVVir_XL5mmb4_B6.raw	1197	17839	7.012724929
7	L1_20210907_BB_HCMVVir_XL5mmb4_B7.raw	1290	17625	7.489361702
8	L1_20210907_BB_HCMVVir_XL5mmb4_B8.raw	2263	21402	10.85412578
9	L1_20210907_BB_HCMVVir_XL5mmb4_B9.raw	2463	23139	10.87341717
10	L1_20210907_BB_HCMVVir_XL5mmb4_B10.raw	2692	23957	11.516467
11	L1_20210907_BB_HCMVVir_XL5mmb4_B11.raw	3830	25046	15.69911363
12	L1_20210907_BB_HCMVVir_XL5mmb4_B12.raw	14	2452	0.57096248
13	L1_20210907_BB_HCMVVir_XL5mmb4_C1.raw	3957	30794	13.23309736
14	L1_20210907_BB_HCMVVir_XL5mmb4_C2.raw	4936	34753	14.57715881
15	L1_20210907_BB_HCMVVir_XL5mmb4_C3.raw	4796	37266	13.37948801
16	L1_20210907_BB_HCMVVir_XL5mmb4_C4.raw	4559	37101	12.76245923
17	L1_20210907_BB_HCMVVir_XL5mmb4_C5.raw	5542	41030	13.98245186
18	L1_20210907_BB_HCMVVir_XL5mmb4_C6.raw	5838	41689	14.61776488
19	L1_20210907_BB_HCMVVir_XL5mmb4_C7.raw	5285	41872	13.21885747
20	L1_20210907_BB_HCMVVir_XL5mmb4_C8.raw	5324	40945	13.61094151
21	L1_20210907_BB_HCMVVir_XL5mmb4_C9.raw	5121	42908	12.41959541
22	L1_20210907_BB_HCMVVir_XL5mmb4_C10.raw	4493	39000	11.99487179

23	L1_20210907_BB_HCMVVir_XL5mmb4_C11.raw	3372	36363	9.614168248
24	L1_20210907_BB_HCMVVir_XL5mmb4_C12.raw	1690	27735	6.20515594
25	L1_20210907_BB_HCMVVir_XL5mmb4_D1.raw	1807	26333	7.067178066
26	L1_20210907_BB_HCMVVir_XL5mmb4_D2.raw	2890	30731	9.849988611
27	L1_20210907_BB_HCMVVir_XL5mmb4_D3.raw	2275	29998	7.820521368
28	L1_20210907_BB_HCMVVir_XL5mmb4_D4.raw	3868	32813	12.04705452
29	L1_20210907_BB_HCMVVir_XL5mmb4_D5.raw	3743	32219	11.97430088
30	L1_20210907_BB_HCMVVir_XL5mmb4_D6.raw	4323	36735	12.05389955
31	L1_20210907_BB_HCMVVir_XL5mmb4_D7.raw	4172	36279	11.69822762
32	L1_20210907_BB_HCMVVir_XL5mmb4_D8.raw	3203	33628	9.676460093
33	L1_20210907_BB_HCMVVir_XL5mmb4_D9.raw	2917	31003	9.56359062
34	L1_20210907_BB_HCMVVir_XL5mmb4_D10.raw	2147	24990	8.771508603
35	L1_20210907_BB_HCMVVir_XL5mmb4_D11.raw	1272	21697	6.042309997
36	L1_20210907_BB_HCMVVir_XL5mmb4_D12.raw	1294	17429	7.860462448

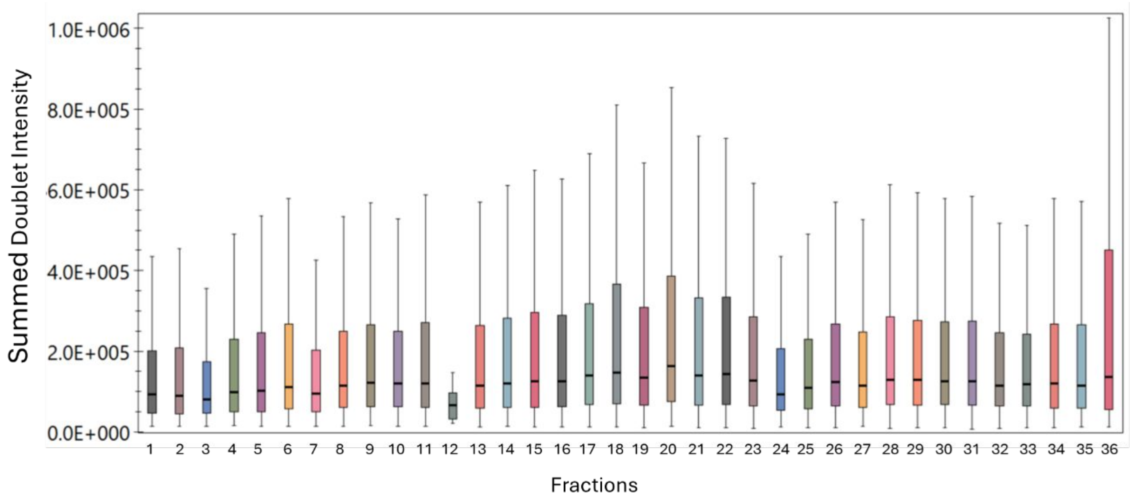

Figure S2. Distribution of the doublets summed intensities in the form of boxplots for each fraction.

3. DSBSO enrichment case study

The dataset used as a case study for enrichment strategies of the DSBSO cross-linker (ProteomeXchange PXD016963) contained five files in total: one with no enrichment and the other four with varying configurations for the enrichment step. Table S2 contains the number of scans which had a doublet identified, as well as the total number of scans and the percentage of scans with doublets for each file analysed in the test done using DSBSO as the cross-linker. Tables

S3 and S4 contain the same information, but for the analysis with DSBU as a cross-linker and with DSBU plus a 1% intensity cut-off, respectively.

Table S2. Summary of the Pair Finder analysis for the enrichment dataset, with DSBSO as the selected cross-linker.

File	#Scans With Pairs	#Scans	Percentage
Lumos_RSLC6_cas9_no_enrich_ctrl.raw	3378	24918	13.55646520587527
Lumos_RSLC6_cas9_3uL.raw	8409	21902	38.39375399506894
Lumos_RSLC6_cas9_6uL.raw	10499	25626	40.97010848357137
Lumos_RSLC6_cas9_12uL.raw	10429	24991	41.731023168340606
Lumos_RSLC6__cas9_24uL.raw	8910	22380	39.812332439678286

Table S3. Summary of the Pair Finder analysis for the enrichment dataset, with DSBU as the selected cross-linker.

File	#Scans with Pairs	#Scans	Percentage
Lumos_RSLC6_cas9_no_enrich_ctrl.raw	729	24918	2.9255959547315196
Lumos_RSLC6_cas9_3uL.raw	725	21902	3.3101999817368277
Lumos_RSLC6_cas9_6uL.raw	977	25626	3.812534145008975
Lumos_RSLC6_cas9_12uL.raw	938	24991	3.753351206434316
Lumos_RSLC6__cas9_24uL.raw	817	22380	3.650580875781948

Table S4. Summary of the Pair Finder analysis for the enrichment dataset, with DSBU as the selected cross-linker and an intensity cut-off of 1%.

File	#Scans with Pairs	#Scans	Percentage
Lumos_RSLC6_cas9_no_enrich_ctrl.raw	432	24918	1.7336864916927524
Lumos_RSLC6_cas9_3uL.raw	468	21902	2.1367911606246004
Lumos_RSLC6_cas9_6uL.raw	630	25626	2.458440646218684
Lumos_RSLC6_cas9_12uL.raw	610	24991	2.4408787163378816

Lumos_RSLC6__cas9_24uL.raw	482	22380	2.153708668453977

4. N-glycopeptides collision energy study

The dataset used for a collision energy study of linear peptides (ProteomeXchange PXD016865) had nine files that differed by the NCE used, from 10% to 50% in increments of 5 units. Figures S3 until S11 show the individual Precursor Signal Ration distribution generated by each of the NCEs.

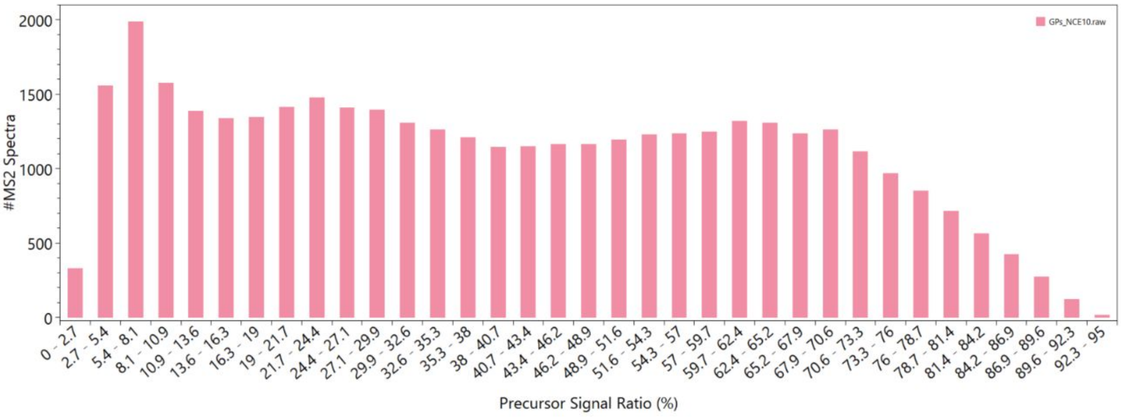

Figure S3. Distribution of the percentage of precursor signal left in the MS2 scans from the experiment with NCE 10%.

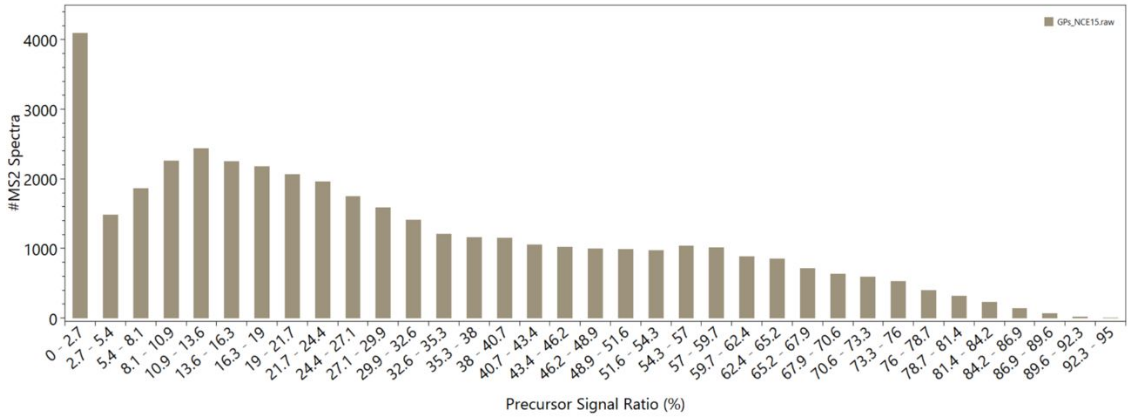

Figure S4. Distribution of the percentage of precursor signal left in the MS2 scans from the experiment with NCE 15%.

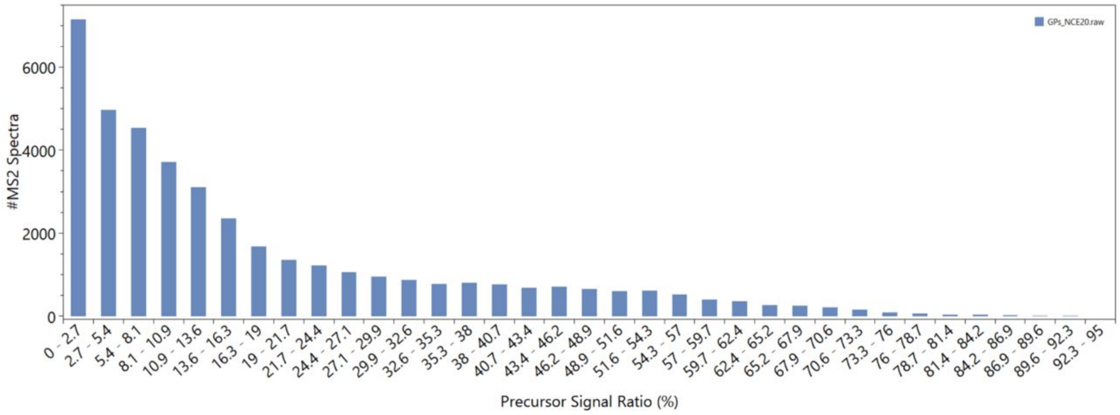

Figure S5. Distribution of the percentage of precursor signal left in the MS2 scans from the experiment with NCE 20%.

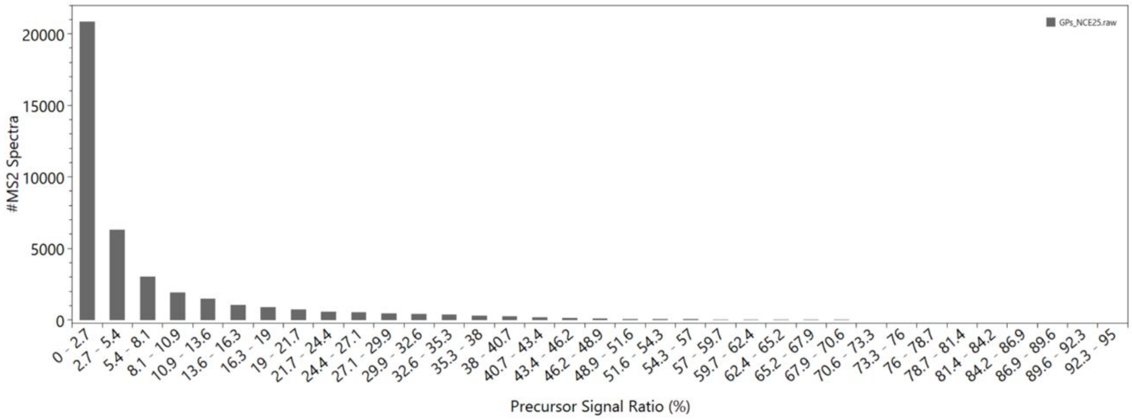

Figure S6 Distribution of the percentage of precursor signal left in the MS2 scans from the experiment with NCE 25%.

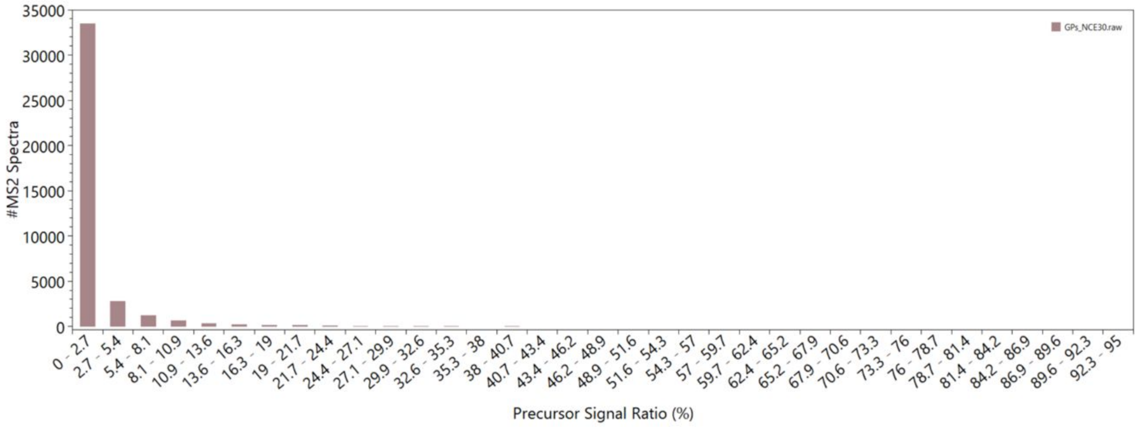

Figure S7. Distribution of the percentage of precursor signal left in the MS2 scans from the experiment with NCE 30%.

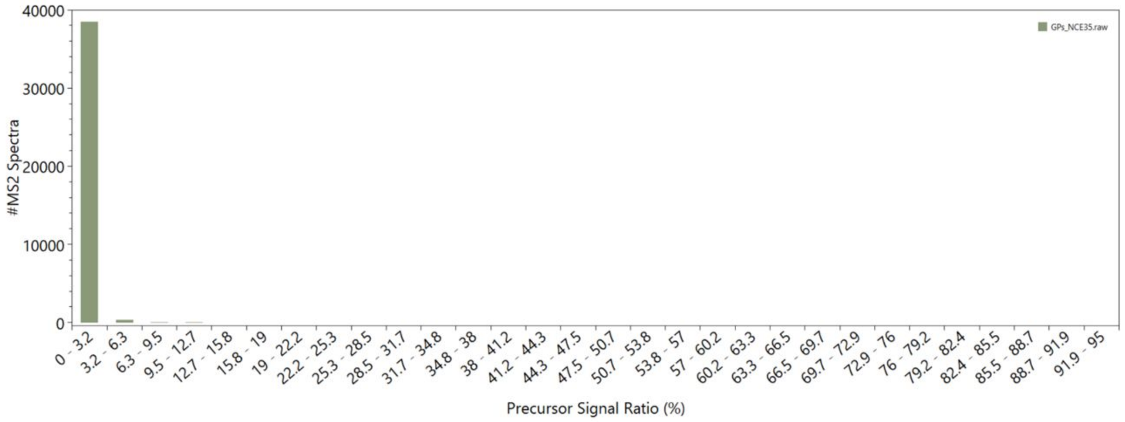

Figure S8. Distribution of the percentage of precursor signal left in the MS2 scans from the experiment with NCE 35%.

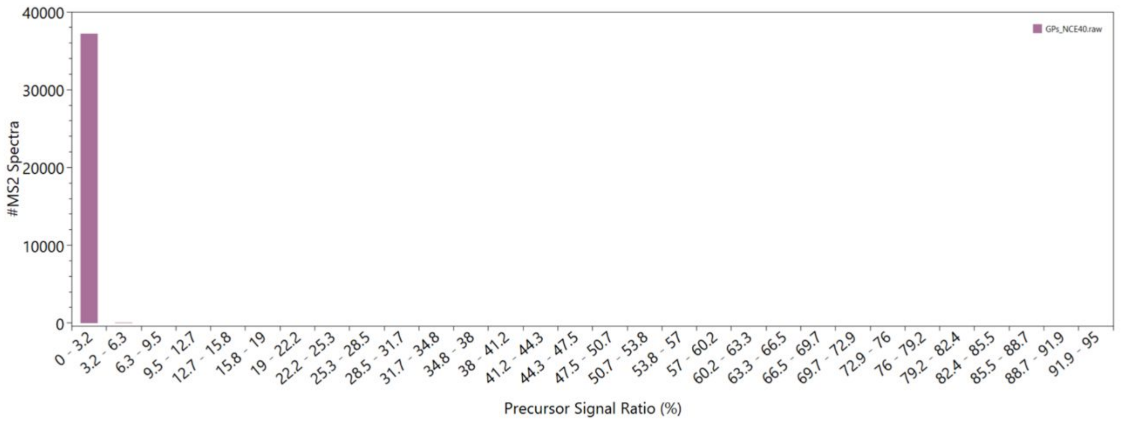

Figure S9. Distribution of the percentage of precursor signal left in the MS2 scans from the experiment with NCE 40%.

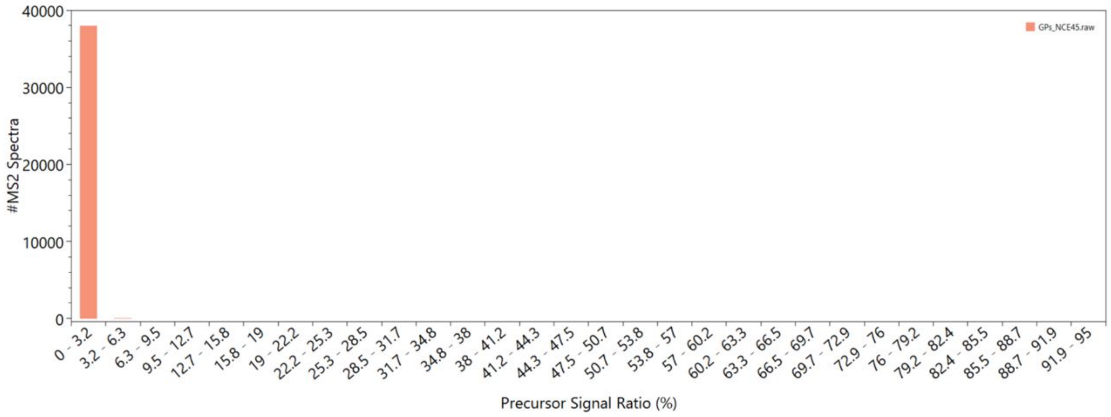

Figure S10. Distribution of the percentage of precursor signal left in the MS2 scans from the experiment with NCE 45%.

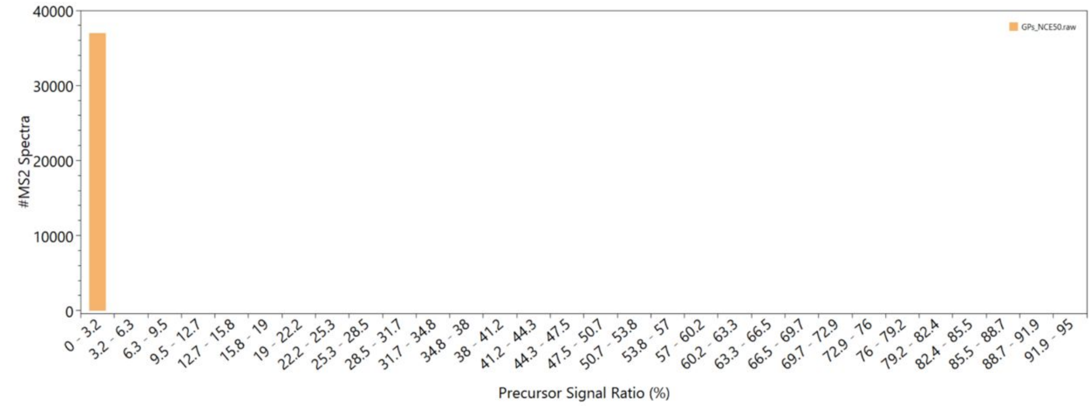

Figure S11. Distribution of the percentage of precursor signal left in the MS2 scans from the experiment with NCE 50%.

5. Cross-linking collision energy case study

A cross-linking dataset was also used as a case study for collision energy analysis. This dataset (ProteomeXchange PXD011861) contained nine different NCEs, from 15% to 39% in increments of 3 units.

Table S5 contains the number of scans which had a doublet identified, as well as the total number of scans and the percentage of scans with doublets for each distinct NCE. Figure S12 displays the distribution of the summed intensity of the doublets for each NCE being analysed.

Figures S13 until S21 show the individual Precursor Signal Ration distribution generated by each of the NCEs.

Table S5. Summary of the Pair Finder results for the different NCEs applied.

File	#Scans with pairs	#Scans	%Pairs
Lumos_RSLC2_BSA_DSSO_Opt_2.raw_15	479	639	74.96087636932708
Lumos_RSLC2_BSA_DSSO_Opt_2.raw_18	531	639	83.09859154929578
Lumos_RSLC2_BSA_DSSO_Opt_2.raw_21	537	639	84.03755868544602
Lumos_RSLC2_BSA_DSSO_Opt_2.raw_24	529	639	82.78560250391236
Lumos_RSLC2_BSA_DSSO_Opt_2.raw_27	498	639	77.93427230046949
Lumos_RSLC2_BSA_DSSO_Opt_2.raw_30	409	639	64.00625978090767
Lumos_RSLC2_BSA_DSSO_Opt_2.raw_33	294	639	46.009389671361504
Lumos_RSLC2_BSA_DSSO_Opt_2.raw_36	192	639	30.046948356807512
Lumos_RSLC2_BSA_DSSO_Opt_2.raw_39	117	639	18.30985915492958

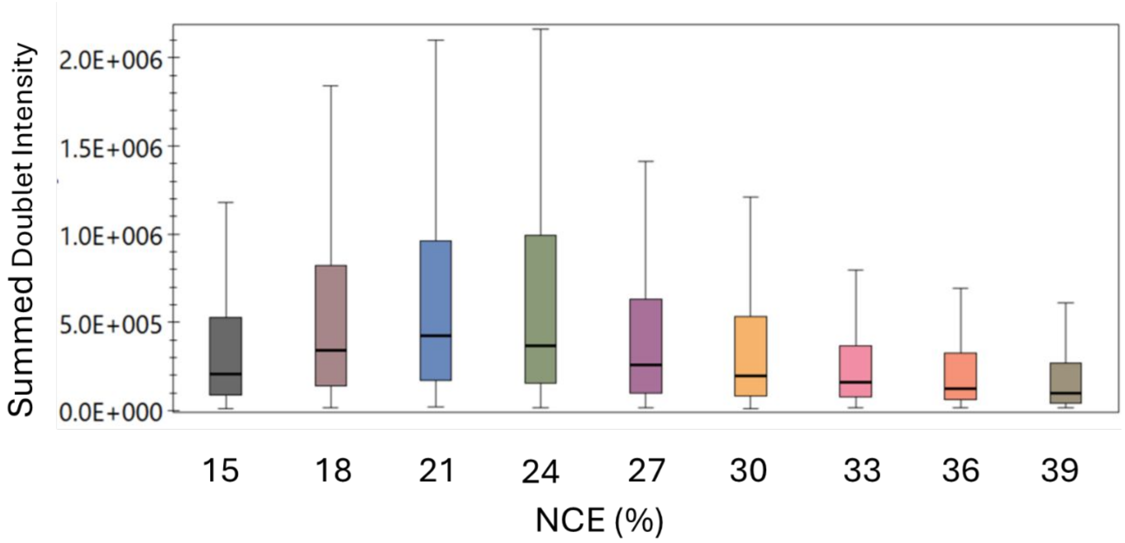

Figure S12. Distribution of the doublets summed intensities in the form of boxplots for each NCE.

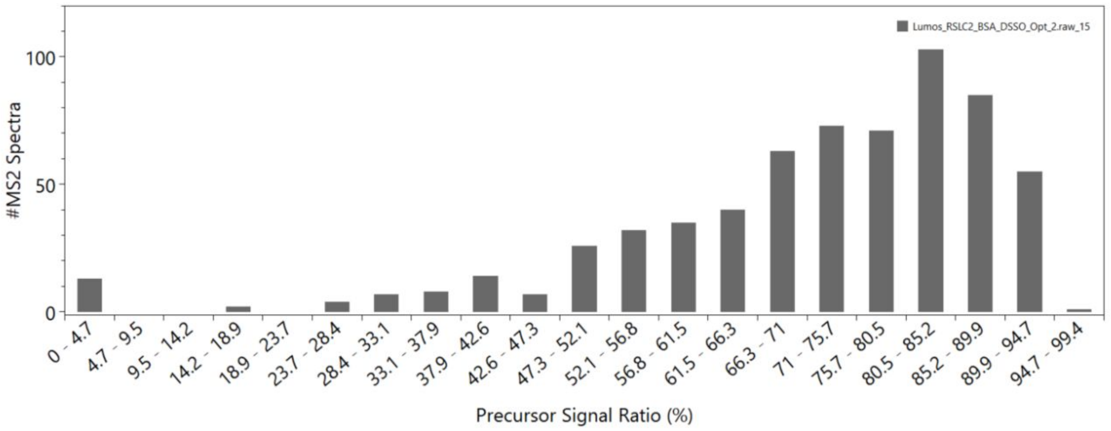

Figure S13. Distribution of the percentage of precursor signal left in the MS2 scans from the experiment with NCE 15%.

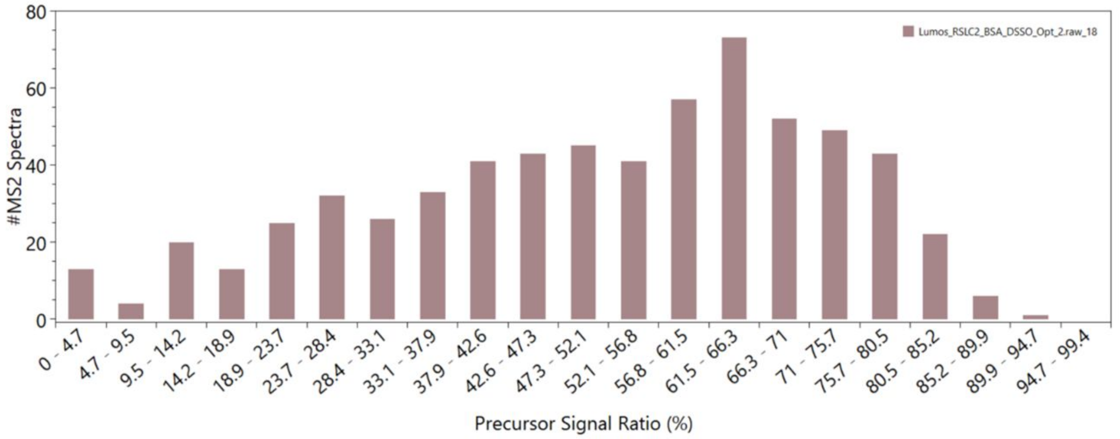

Figure S14. Distribution of the percentage of precursor signal left in the MS2 scans from the experiment with NCE 18%.

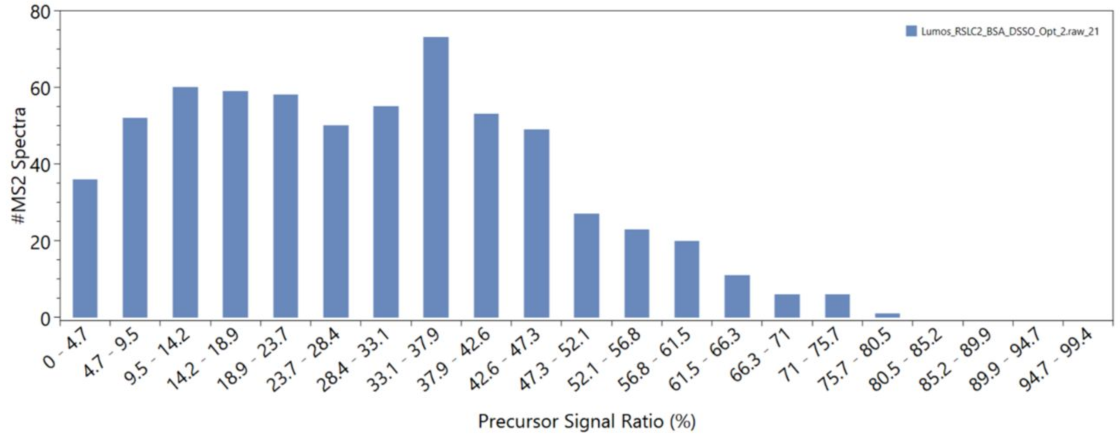

Figure S15. Distribution of the percentage of precursor signal left in the MS2 scans from the experiment with NCE 21%.

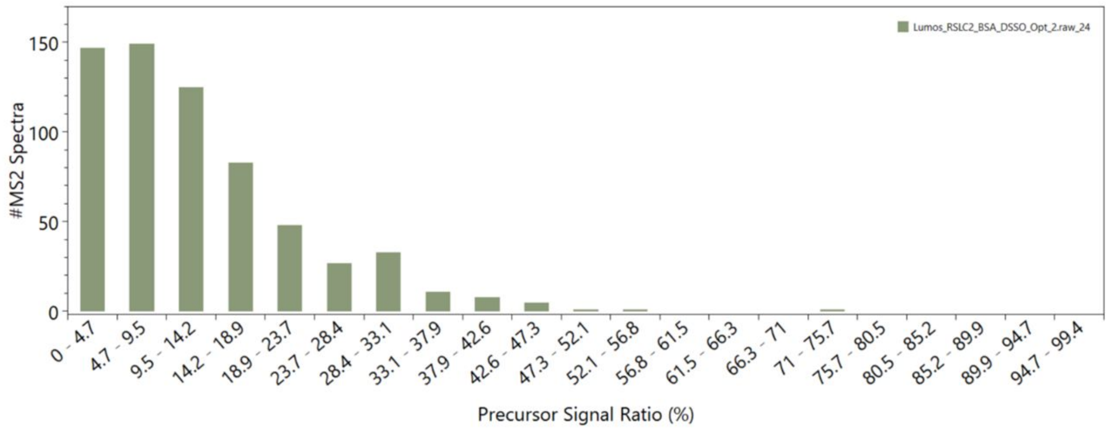

Figure S16. Distribution of the percentage of precursor signal left in the MS2 scans from the experiment with NCE 24%.

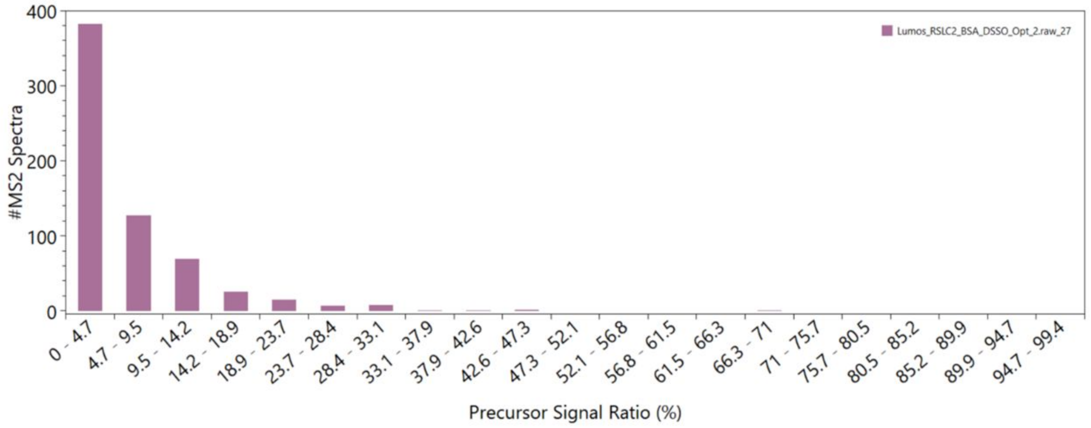

Figure S17. Distribution of the percentage of precursor signal left in the MS2 scans from the experiment with NCE 27%.

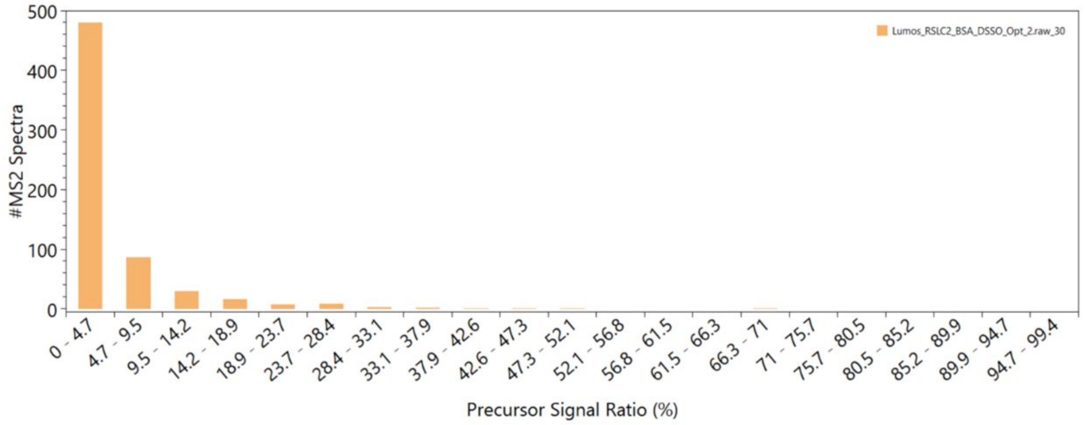

Figure S18. Distribution of the percentage of precursor signal left in the MS2 scans from the experiment with NCE 30%.

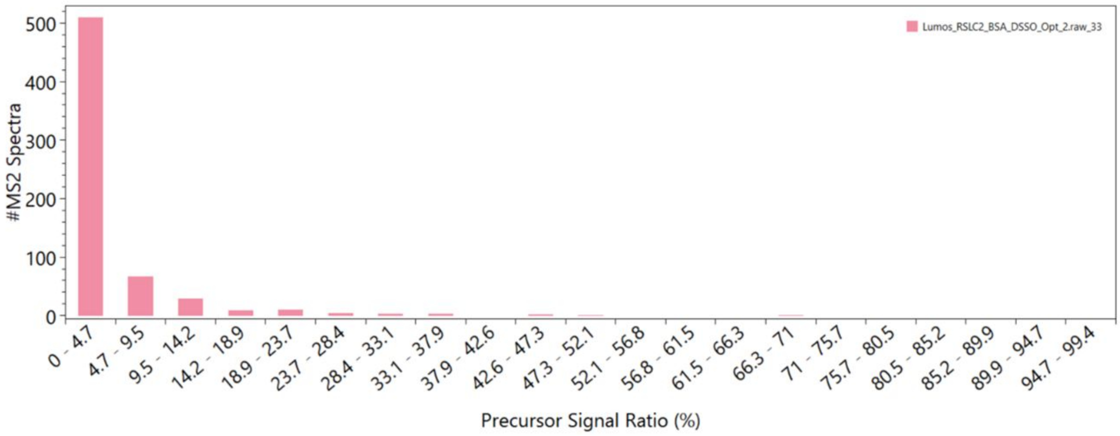

Figure S19. Distribution of the percentage of precursor signal left in the MS2 scans from the experiment with NCE 33%.

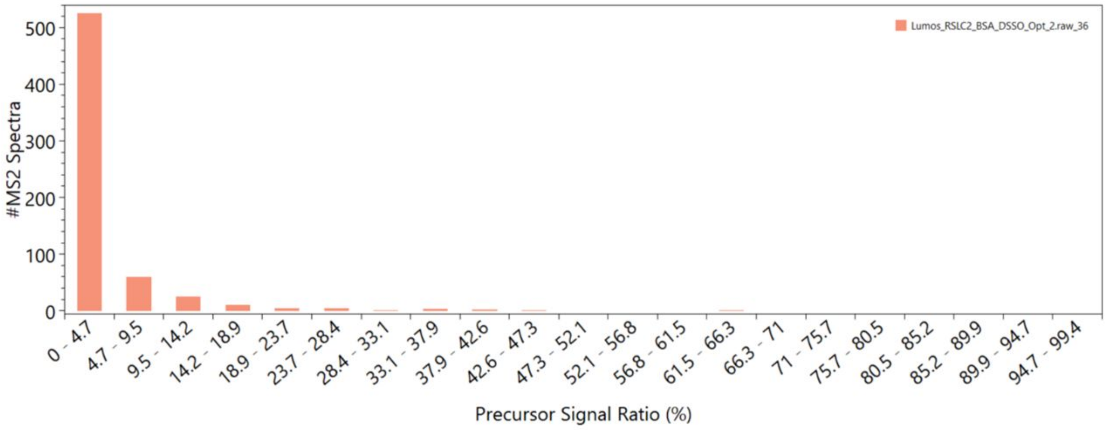

Figure S20. Distribution of the percentage of precursor signal left in the MS2 scans from the experiment with NCE 36%.

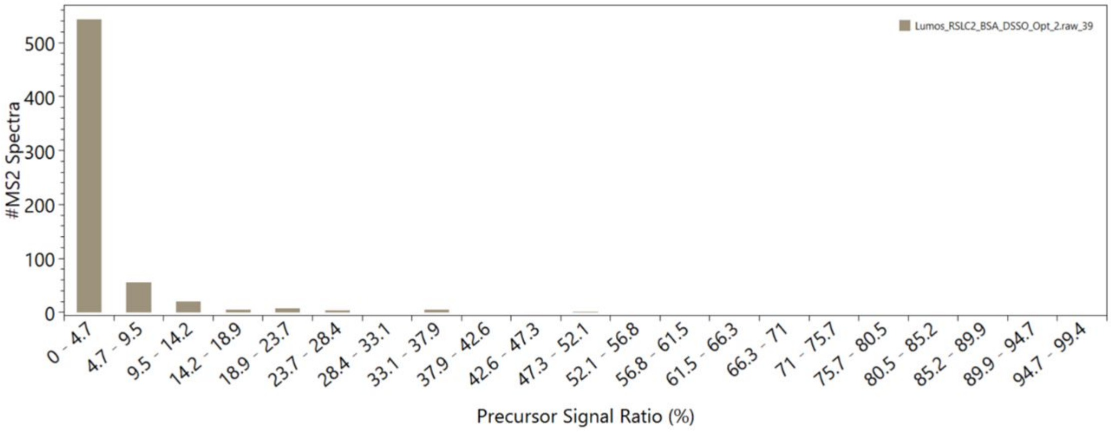

Figure S21. Distribution of the percentage of precursor signal left in the MS2 scans from the experiment with NCE 39%.

6. RawVegetable 2.0's General Updates

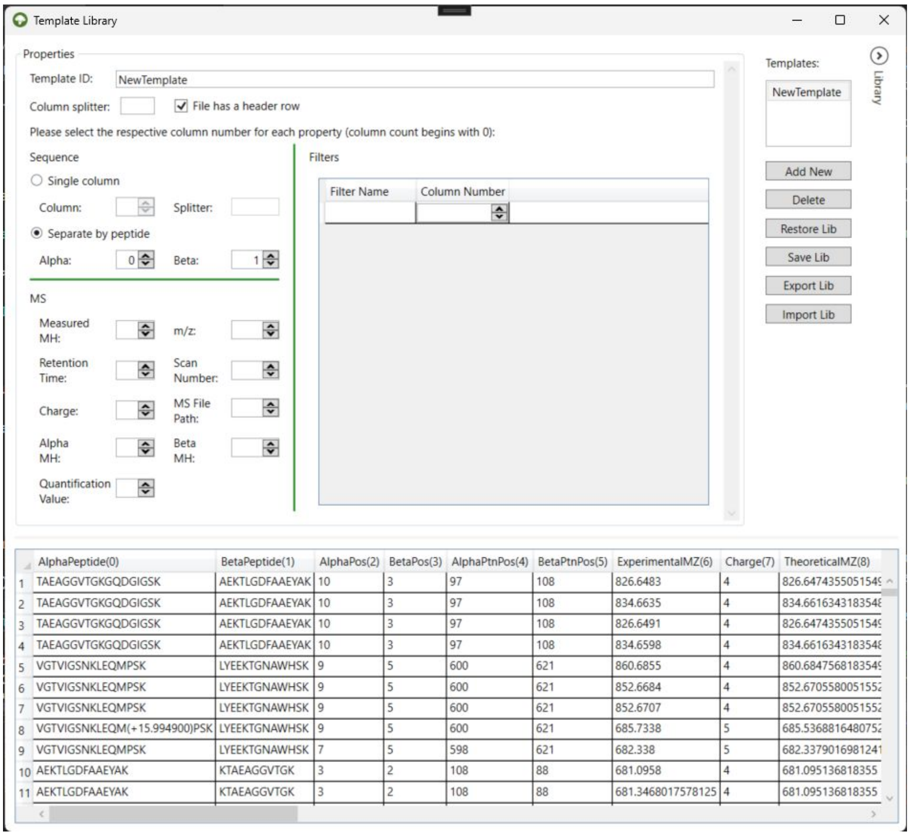

Figure S22. Graphical user interface for loading identification results. The software now features a versatile reader that supports both *.csv and *.txt file formats.

REFERENCES

(1) Savitzky, A. A Historic Collaboration. *Anal. Chem.* **1989**, *61* (15), 921A-923A. https://doi.org/10.1021/ac00190a744.

(2) Savitzky, Abraham.; Golay, M. J. E. Smoothing and Differentiation of Data by Simplified Least Squares Procedures. *Anal. Chem.* **1964**, *36* (8), 1627–1639. https://doi.org/10.1021/ac60214a047.
